# Dementia During the COVID-19 Pandemic: Experiences From Clinicians, Patients, and Caregivers in Switzerland

**DOI:** 10.1177/23337214231164911

**Published:** 2023-03-30

**Authors:** Eileen Neumann, Lisa Ballmer, Olivia Studhalter, Nicole Schmid, Hans H. Jung

**Affiliations:** 1 University Hospital Zurich, Switzerland; 2 University of Zurich, Switzerland

**Keywords:** dementia, pandemic, experiences, qualitative, Switzerland

## Abstract

**Background:** The COVID-19 pandemic had a significant impact on the Swiss health care system, affecting especially vulnerable people, such as patients suffering from dementia. The purpose of this study was to investigate the challenges experienced by dementia patients, their carers, and clinicians during the pandemic in Switzerland. **Methods:** An online survey was sent to all memory clinics in the German speaking part of Switzerland. Patients diagnosed with dementia and their carers were recruited for semi-structured telephone interviews at the memory clinic of the University Hospital Zurich. **Results:** A total of 28 clinicians, 17 carers, and seven patients participated in this study. According to the clinicians, all aspects of clinical work were affected by the pandemic. Carers did not perceive a significant role of the pandemic in the disease progression of the patients, despite many challenges faced. Patients described a high level of conscientiousness during the pandemic. Recommendations for future scenarios were provided from all groups. **Conclusion:** In order to increase the systemic resilience of the Swiss health care system, it is important to consider the experiences and recommendations of vulnerable groups and health care professionals for future public health measures and policies.

## Introduction

Dementia is a clinical syndrome resulting from different underlying diseases, for example, Alzheimer’s disease or vascular dementia. The main symptom of dementia is progressive cognitive decline, which goes beyond the average effect of aging and interferes with daily activities of living. Worldwide, dementia is the seventh leading cause of death among all illnesses (World Health Organization, 2022a). In Switzerland, dementia ranks now as the second most common cause of death, outnumbering stroke, cancer, respiratory diseases, and accidents (World Health Organization, 2022b). Managing the increasing numbers of dementia patients is an enormous challenge for the health care system in Switzerland. Even before the COVID-19 pandemic put an additional strain on the health care system, only about half of the dementia patients in Switzerland had a diagnosis confirmed by a specialist (Alzheimer Schweiz, 2021). At the end of January 2020, the World Health Organization (WHO) declared the outbreak of the novel coronavirus (SARS-CoV-2) a public health emergency of international concern. Since then, the Swiss government has implemented various measures and ordinances, including two national lockdowns (with a total length of 83 days), social distancing, quarantine, and isolation rules, aiming to reduce the infectious spread of the deadly virus (Federal Office of Public Health, 2020). While the effectiveness of these interventions has been studied intensively (Perra, 2021), the scale of adverse health effects, especially in the long-term, is only beginning to emerge. We already know that negative psychological outcomes, such as depression, anxiety, or stress are associated with social isolation measures during the pandemic, even in previously healthy people (Brooks et al., 2020). Further, detrimental effects on medical practice, such as diagnostic delays and a decrease in therapy and follow-up visits have been reported (Perra, 2021). Already at the end of 2020, European dementia specialists from six countries (including Switzerland) reported a significant toll on their patients due to isolation (Burns et al., 2021). In line, mental health issues in Swiss caregivers of people with dementia were found to be elevated during the first wave of the pandemic (Messina et al., 2022). To the best of our knowledge, there are currently no qualitative studies on the challenges experienced by dementia patients, their carers, and clinicians during the COVID-19 pandemic in Switzerland.

## Methods

### Study Design

This study adopted a mixed methods design with both quantitative and qualitative approaches. For the first part, an online survey was designed and sent to all (37) memory clinics in the German speaking part of Switzerland. The survey included both closed as well as open-ended questions about the clinicians’ experiences during the pandemic. For the second part, a semi-structured telephone interview about the patients’ and carers’ experiences during the pandemic was designed. Further information from carers and patients was collected based on questionnaires (see *Measures*). Ethics approval was waived by the ethics committee of the canton Zurich (BASEC-Nr. 2022-00502).

### Subjects and Recruitment

Patients and carers were recruited from the memory clinic at the University Hospital Zurich based on the criteria shown in Table 1.

**Table 1.** Selection Criteria for Patients and Carers.

Inclusion criteria	Exclusion criteria
• Patients registered at memory clinic (consultation since 01/2019) • Diagnosis is either: º Alzheimer’s disease º Lewy body dementia º Frontotemporal dementias (including PSP and CBD) º Vascular dementia º Parkinson’s disease dementia º Dementia of unknown etiology • Primary caregiver available for the study • Patient and carer speak and understand German • Patient and primary caregiver were informed, and both gave his/her consent to the study	• Dementia diagnosis not listed in inclusion criteria • Documented objection to participation • Patients not able to perform telephone interview as assessed during screening interview by psychologist (i.e., insufficient communication abilities)

Patients and carers were contacted twice: first to inform them about the study and to get their consent to schedule an appointment for the interview and a second time for data collection (at least 1 week later). Withdrawal from the study at any time without reason or consequences was guaranteed. Informed consent was given verbally via telephone both during the initial screening interview and before the start of data collection.

### Measures

Sociodemographic data was collected from clinicians, patients, and carers. Clinical data and behavioral/neuropsychiatric symptoms based on the *Geriatric depression scale* (GDS) and the *WHO Quality of Life-question 9* (WHOQoL) was acquired from patients. The *Neuropsychiatric Inventory-Questionnaire* (NPI-Q), and the *Depression, anxiety, and stress scale-21 items* (DASS-21) were conducted with the carers.

### Procedure

The survey was filled out between 05 and 07/2022. Telephone interviews were conducted between 06 and 08/2022 in either Swiss or Standard German. The interviews were recorded to prevent data loss and subsequently transcribed and translated into Standard German. The first version of the transcription was prepared by OS. The transcription was read and verified by EN, based on the audio files. For the preparation of the manuscript, results were translated into English.

### Data Analysis

Conventional content analysis was used for open-ended questions: data was grouped around central, recurring thoughts to form overarching themes. For general closed questions, answers were counted and summarized. If limited information was available or the majority of answers went in a similar direction, answers were also summarized. Non-relevant information (e.g., off-topic conversation) was left out of the analysis. Due to the large amount of available data, only the most relevant results are reported in this manuscript. Quantitative data was analyzed using SPSS Version 28.

## Results

### Survey

#### Sample Characteristics of Clinicians

Fifteen out of the 37 contacted memory clinics responded to our survey (response rate 41%), and a total of 28 clinicians filled out the survey. The sociodemographic characteristics of clinicians are summarized in Table 2. Noteworthy, about 50% of all clinicians had been working at the respective memory clinic for more than 5 years, at the time of the survey.

**Table 2.** Sociodemographic Characteristics of Clinicians.

Characteristic	Value
Age (average years; range)	45 (27–67)
Nationality (*N*)	Swiss 14
	German 10
	Austrian 3
	Mixed nationalities 1
Gender (% female/male)	61/39
Occupation (*N*; %)	Neuropsychologists 10 (36)
	Medical doctors 6 (21)
	Leading physicians/psychologists 12 (43)

#### General Questions

**Figure 1.**
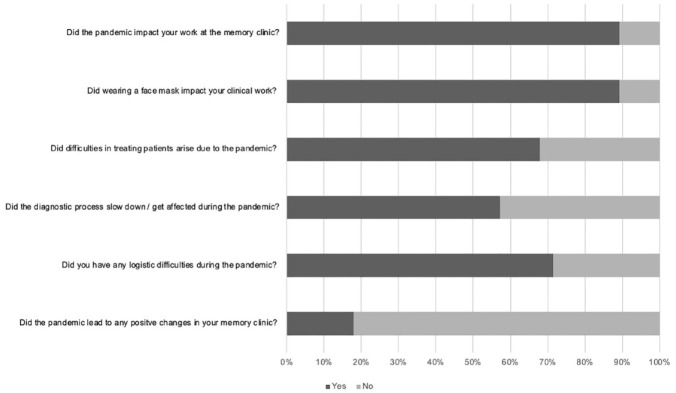
Responses to general questions by clinicians.

Answers to the general closed questions are shown in Figure 1.

#### Follow-Up Questions

##### Clinicians’ Work

Clinicians reported an impact of the pandemic on several aspects of their work at the memory clinic. First, patient interaction changed: there were more remote consultations and less direct contact with patients; consultations were adjusted (e.g., less participants such as family members; increased physical distance; physical, neurological, and neuropsychological examinations were shortened); and communication was more difficult due to mandatory face masks and protective shields. Second, organizational issues emerged: appointments were canceled by patients or by the clinic (in some cases closed for several weeks); long-term scheduling and planning became more difficult and an increase in bureaucracy and documentation was reported. Third, interactions within the clinicians’ team changed due to difficulties with interdisciplinary exchange (e.g., the social support team), less communication within the team in general (e.g., due to mandatory home office, sudden changes in the rotation plan, or remote trainings or meetings), reduced staff due to sickness or taking over other (COVID-19 related) responsibilities within the hospital, and the general psychological burden of the pandemic on team members. Finally, factors relating directly to the patient’s well-being were reported: unaccompanied patients due to the hospital rules; cancelations of cognitive trainings and support groups, and the psychological burden of patients and their families (e.g., aggravation of symptoms due to restrictions).

##### Protective Face Masks

Clinicians described four general issues with regards to the impact of face masks on their work. First, physical problems (for both the patient and the clinician), for example, pain behind the ears, tiredness, headaches, feeling hot, and a decrease in fluid intake during the day. Second, challenges regarding communication and bonding with patients: lip-reading was not possible for patients with hearing impairments or for non-native speakers; non-verbal and emotional cues were reduced and the physical barrier between patients and clinicians created a less personal and less emotional connection. Third, the assessment of neurological aspects of the face was described to be more difficult with the face masks (e.g., facial expression or buccofacial apraxia). Finally, an interruption of the clinical routine was reported, due to numerous reminders for the patient to wear the mask or to place the mask correctly as well as discussion with patients and relatives regarding the necessity of the measures.

#### Challenges in the Diagnostic Process

Clinicians were asked to rank which factor had the greatest negative impact on the diagnostic process during the pandemic. Most frequently (nine times), clinicians stated that the ambiguous cognitive decline of patients was the greatest difficulty during the diagnostic process, that is, it was not clear whether their cognition was deteriorating due to the social isolation or due to a neurodegenerative process. This was followed by cancelations of appointments by patients or by the clinic; missed recognition of symptoms by relatives during the lockdown; and finally, staff shortage due to sickness or newly assigned rules.

#### Challenges in the Treatment Process

Four reasons for challenging treatment conditions during the pandemic were outlined by clinicians. First, availability of symptomatic treatment (e.g., psycho- or occupational therapy), and external support (e.g., day clinics) was reduced in general. Second, significant delays occurred due to canceled appointments, latencies of referrals, and longer waiting lists for diagnostic measures and appointments at the memory clinic. Third, communication between clinicians, patients, and carers was reduced and information about the diagnosis or the planned treatment was not transferred sufficiently. Finally, the psychological stress of the pandemic on the patient, for example, social isolation and loneliness, fear of infection or confusion about the ongoing situation, affected the treatment process as well.

##### Logistic Difficulties

Numerous logistical difficulties were reported: transportation to the memory clinic, organization of COVID-tests or certificates for patients and relatives, checkpoints at entrances, new entrances, access to parking, limited physical space for testing, and frequently changing rules in the hospitals, for example, different forms needed for access. Some hospital rules did not allow accompanying persons, which led to delays, no-shows, and missing information from caregivers.

##### Positive Changes

Three themes emerged, when clinicians were asked about positive changes at their memory clinic. First, the possibility to set up virtual conversations or consultation (e.g., to include relatives living further away). Second, an increase in solidarity and appreciation within the team; and third, an increase in effectiveness of internal processes, for example, determining the necessity of a personal appointment.

##### Future Recommendations

The following recommendations for future pandemics were provided by clinicians: first, the importance of direct (also physical) contact with relatives was stressed, to ensure mobility and mental health of patients. Moreover, unchanged access to therapies and external assistance was advised. The importance for patients with dementias of having early access to vaccinations and protective material as well as specific recommendations was further highlighted. Additionally, reinforcing remote consultations and programs for social activities (e.g., online or via telephone) were demanded. Finally, the self-determination of patients, for example, whether or not to wear protective masks, was emphasized.

### Telephone Interviews

#### Sample Characteristics of Patients and Carers

Twenty telephone interviews were held (13 with carers, 4 with both carers and patients, 3 with patients). Table 3 summarizes the sample characteristics at the time of the interviews. The majority of patients was diagnosed with Alzheimer’s disease and was of Swiss nationality.

**Table 3.** Sociodemographic and Clinical Data of Patients and Carers.

	Patients	Carers
Age, mean (range)	77 (64–85)	68 (34–91)
Gender (% female/male)	60/40	47/53
Nationality (*N*)	Swiss (18)	Swiss (15)
	Kosovan (1)	Austrian (1)
	Italian (1)	Italian (1)
Diagnosis (*N*; %)	Alzheimer’s disease 13 (65)	
	Vascular dementia 2 (10)	
	Mixed dementia 2 (10)	
	Atypical Parkinson’s 1 (5)	
	Unknown 2 (10)	
Relationship carer/patient (%)	Spouse (71)	
	Child (29)	

#### Behavioral and Neuropsychiatric Scores

The behavioral and neuropsychiatric scores of patients at the time of the interview are summarized in Table 4. On average, carers reported neuropsychiatric symptoms on 5 to 6 domains (out of 12), with a moderate to severe severity and mild distress for the carers. The average score of about 4 (out of eight activities of daily living) on the IADL corresponds to about 50% dependence. On self-rated questionnaires, patients showed on average no signs of depression and described their quality of life as good (three patients did not provide an answer to this question), even though they were dependent on somebody else with about 50% of their daily activities of living.

**Table 4.** Behavioral and Neuropsychiatric Scores of Patients.

Questionnaire	Mean (*SD*)	*N*
NPI-total	5.8 (2.2)	17
NPI-severity	2.4 (0.7)	17
NPI-distress	2.2 (1.1)	16
IADL-total	3.8 (2.6)	15
GDS-total	1.6 (1.7)	7
WHOQoL-9	2 (0)	4

*Note.* NPI = Neuropsychiatric Inventory; IADL = Instrumental Activities of Daily Living; GDS = Geriatric Depression Scale; WHOQoL = World Health Organization Quality of Life.

Carers themselves scored on average within the normal range on symptoms relating to depression, anxiety, and stress (DASS-21).

### Patients’ Perspective

#### General Effect of the Pandemic on the Life of Patients

Three general themes emerged when analyzing the effect of the pandemic on the life of patients. The first theme was labeled *conscientiousness*: the majority of patients described a sense of duty to follow the new rules and restrictions, including getting the vaccination, social distancing, and staying informed. One patient commented that “if you follow the rules, everything is alright” and another patient described getting the vaccination as “a contribution to society.” A subtle criticism of people not following the rules was also present, for example, one patient commenting that “there are enough people, who are not careful.” The second theme was labeled *unchanged state*: in contradiction to the above-described awareness of new regulations, the majority of patients also described that their lives did not change during the pandemic. One patient even said that she “did not notice the pandemic,” while later stating that she adhered to the new rules. The third theme was named *fear*: negative thoughts and fearful emotions were mentioned by two patients, one of which was battling cancer on top of dementia. The pandemic was described as a “dangerous matter,” especially for those who had suffered a lot already. The importance of not developing a “phobia” of the disease and staying as objective and rational as possible was stressed by another patient.

#### Effect of the Pandemic on the Physical and Mental Health of Patients

Most patients reported that they did not observe any effect or only a small effect of the pandemic on their physical health. If an effect was reported, it was related to minor bodily pains due to a decrease in mobility or activity (e.g., taking the stairs less frequently). The majority (five out of seven) patients reported no effect of the pandemic on their mental health. One patient reported a negative effect, that is, being more depressed and thinking more about the “outside world.” One patient’s statement was unclear and not leaning in either direction.

#### Future Recommendations

Overall, patients were content with the regulations during the pandemic and did not wish for different rules or restrictions. Three aspects were raised, when considering the needs of people living with dementia: first, the importance of direct social contact for example, meeting family members or friends. One patient described her difficulties of staying in touch with people via telephone, due to her hearing aids and language disabilities. Second, it was requested that the rules should be followed by everyone, especially less vulnerable groups. Third, a concern about too many different opinions within the closest circle of family and friends was raised, as this felt counterproductive.

### Carers’ Perspective

#### General Questions

**Figure 2.**
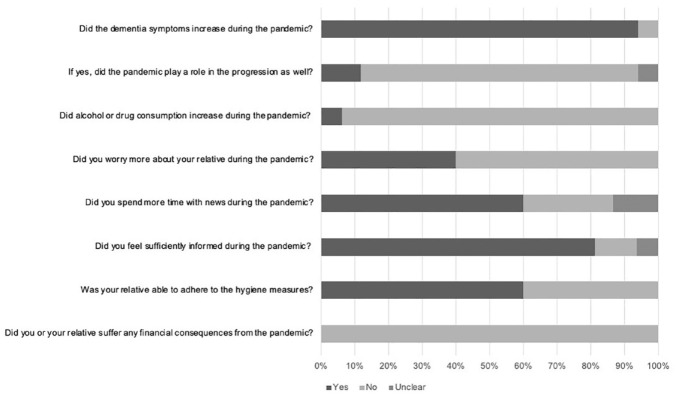
Responses to general questions by carers.

Answers to general closed questions are summarized in Figure 2. Importantly, most carers reported a progression of dementia symptoms during the pandemic but did not see a significant role of the pandemic in the progression. When directly comparing patients’ and carers’ perception of the progression of symptoms during the pandemic (these data were available for four patients), the answers did not align: only one out of four patients stated that there might have been a progression whereas three of their carers clearly saw a progression during the pandemic. Even though 13 carers indicated that they did feel sufficiently informed overall during the pandemic, six carers further elaborated, that they were overwhelmed with the available information or felt that there was too much unnecessary information in the media, sometimes fueling fear and panic. As one carer said: “it was difficult to filter out what was correct and what was not.”

#### Effect of the Pandemic on the Physical and Mental Health of Patients

The majority of carers (12 out of 15) reported no effect of the pandemic on the physical health of patients. Five carers talked about how other effects, such as the progressed dementia, other diseases, medication side effects or the general mindset of patients were so prominent, that the pandemic had no additional influence. For example, one carer said: “she is always stubborn and doesn’t want to -go out for a walk-. She always finds a reason not to.” One carer reported a negative effect of the pandemic on the physical health of his wife, he said it was “one more obstacle” for activities. Two carers described a positive effect of the pandemic on the physical health of patients, mainly due to a calmer daily routine and more time spent on walking. The majority of carers (nine out of 16) described a negative effect of the pandemic on the mental health of patients. For two patients, the negative effect was mainly noticeable in the beginning of the pandemic. Most frequently, loss of activities, loneliness, and boredom was mentioned. Three carers further described an increased anxiety, either unspecific or a fear of getting infected. One carer said: “he lives with the fear, even now that things have calmed down, it is still an issue for him.” Of particular note, one carer described the psychological burden of his wife having to say goodbye to her dying father through a protective glass shield. Seven carers described no effect of the pandemic on the mental health of patients. Three carers of this group further elaborated, that patients did not realize or did not fully understand the pandemic, while two carers pointed out, that the patient was already isolated or depressed before the pandemic.

#### Effect of the Pandemic on the Mental Health of Carers

The vast majority of carers (11 out of 16) reported no or only a small influence of the pandemic on their mental health. The general attitude appeared to be pragmatic and realistic, for example, three carers used the phrase “making the best out of the situation.” The missed activities and the inconvenience of wearing masks and following the recommendations and rules was mentioned most frequently among this group, as aspects with potential effect on their mental well-being.

Three carers reported a significant negative effect of the pandemic on their mental health, including an increase in worrying and a decrease in patience. Two of these carers stressed the importance of being able to have a “timeout” and putting physical distance between themselves and their home, for example, going for a walk by themselves. Two carers reported a positive effect of the pandemic on their mental health, due to their less stressful schedule.

#### Future Recommendations

The most frequent recommendation from carers was centered around the isolation of patients during the pandemic, especially in nursing homes. The direct contact to other people was described as “essential,” and one carer recommended that social networks for older people should be strengthened, regardless of the pandemic. Especially for doctors and home carers, caregivers recommended spending more time to explain the ongoing situation and necessary measures to the patients. One carer said: “with dementia it is difficult anyway, if something new happens and suddenly you don’t understand why you have to wear a mask everywhere.” Two carers expressed their wish for more solidarity within the country during the pandemic and less resistance against necessary measures. Finally, home vaccinations as an option were recommended, to decrease the logistic difficulties.

## Discussion

### Clinicians’ Experience of the Pandemic

Our results demonstrated that based on the clinician’s experience, all aspects of clinical work (early logistical aspects, diagnostics, and treatment) were affected by the pandemic. This finding is in line with previous work (Bianchetti et al., 2022). However, the specific factors, such as patient communication or adjustments of clinical assessments, were not previously identified. It is important to consider clinician’s recommendations, such as the continued direct (also physical) contact with close caretakers or relatives, for possible similar future exceptional circumstances.

### Patients’ Experience of the Pandemic

The high degree of conscientiousness among dementia patients during the pandemic as reported in our study is in line with a previous finding by Zimmermann et al. (2021) who described solidarity or a perceived moral obligation as a motivation to wear face masks among patients. A clear paradox between being aware of new rules, such as face masks, and a feeling of an unchanged state became apparent among some dementia patients. This paradox may reflect how patients are still able to learn simple, recurring procedures, while they have difficulties with more complex cognitive tasks, such as apprehending an ongoing global situation. Fear of infection has been reported widely among caregivers of people living with dementia (Cohen et al., 2020; Vaitheswaran et al., 2020), yet, in our study only two patients reported fearful emotions with regards to the pandemic. Possibly, this may also relate to patients’ difficulties of fully comprehending their situation. In contrast to the clinicians’ experience, most patients in our study did not perceive an effect of the pandemic on their physical or mental health, and most carers agreed on the physical aspect. However, with regards to mental health, the results were more diverse: while a little more than half of the carers clearly observed a negative effect of the pandemic on the mental health of their relatives, the rest of the carers did not report such an influence. According to the carers who did not see a negative effect, this was due to the patients’ inability to recognize the changes around them as well as their isolated situation pre-pandemic. It could be argued that this result contrasts with findings by Boutoleau-Bretonnière et al. (2020) who described a greater incidence of neuropsychiatric symptoms during confinement in patients with lower cognitive functioning. However, Boutoleau-Bretonnière’s result is based on an objective clinical assessment and not the subjective perception of carers. Furthermore, the data was acquired during the peak of the pandemic, whereas our data was collected more than 1 year after the start of the pandemic. The result that more carers perceived a progression of dementia symptoms during the pandemic than patients is not surprising, since unawareness of symptoms or symptom progression in dementia patients has been well described (Aalten et al., 2005). In line with the clinician’s recommendation, patients also described the importance of direct social contact for their well-being. Overall, the patients’ recommendation for an adherence to the rules by all citizens fits the above-described theme of solidarity and conscientiousness.

### Carers’ Experience of the Pandemic

A novel finding of this study is that according to the carers’ experience, the pandemic did not play a significant role in the progression of the disease. This finding suggests that from the carers’ point of view, the progression of dementia is relatively independent from external factors, such as the challenging situation faced during the lockdown. Alternatively, it could mean that carers do not perceive the influence of external factors on the patients as significant in general. A further novel finding is that most carers did not experience a deterioration of their mental health during the pandemic, despite an increase in worrying about the patients, spending more time informing themselves as well as the general daily challenges, such as patients having difficulties with following the recommended hygienic measures. This finding is further supported by normal scores on symptoms of depression, anxiety, and stress as well as an on average mild carer distress by patients’ neuropsychiatric symptoms. As raised by clinicians and patients, the recommendation to limit isolation of patients was further supported by carers. Social isolation and loneliness are of special importance when discussing dementia. There is evidence, that social isolation is significantly associated with poor cognitive function in later life (Evans et al., 2019). Further, perceived loneliness, as a more subjective factor compared to social isolation, is a known risk factor for dementia, especially Alzheimer’s disease (Sundström et al., 2020). Considering both the scientific knowledge as well as the consistent advice from clinicians’ and people affected by dementia for future scenarios is important. The patients’ theme of solidarity re-occurred among the carers as well, wishing for less resistance against recommended measures from the population. This is consistent with research describing increased stress and frustration among a group of British carers of people living with dementia, when being confronted with hygienic misconduct (Hanna et al., 2022). Finally, carers’ dissatisfaction with the information provided by official institutions has been described before Zimmermann et al. (2021, p. 5) and needs to be addressed.

### Strengths and Limitations

This study seeks to understand a novel situation based on three different angles: the professional, the direct personal, and indirect personal point of view. Certain limitations of this study are directly linked to our sample. First, some of the patients as well as some of the carers had difficulties with understanding the questions and/or being able to provide an answer. This is partly due to the dementias, but also due to age-related changes such as hearing difficulties or diseases. Second, the majority of patients was diagnosed with Alzheimer’s disease, thus, it is unclear, if patients suffering from other types of dementia experienced the pandemic differently. Third, the sample consists of mainly Swiss nationals. Since some results are connected to cultural aspects, for example, solidarity as an important value in the Swiss culture (Biller-Andorno & Zeltner, 2015), the transferability of our results on other countries is questionable. As our study was conducted, the pandemic had already passed its’ peak. It was challenging for all participants to describe and remember the past experiences correctly, especially since their memory functions are impaired and the situation had changed over time. Furthermore, a recency effect (recalling the most recent events more accurately) as well as a rosy retrospection bias (a predisposition to view the past more positively) can be expected. Thus, it should be assumed that the actual experiences were a bit more negative than reported. Finally, the sample was limited to patients and carers from the University Hospital Zurich, due to the available resources of the study.

### Future Directions

The impact of the pandemic on society and the health care system is unprecedented. However, based on mathematical models (Marani et al., 2021), there is a high probability of experiencing a similar pandemic within the next decades. Thus, it is important to learn from the current situation in order to improve future policies, especially when considering vulnerable groups such as dementia patients and their caregivers. There is an urgent need for more studies that consider the experience and recommendations of the people strongly affected by the policies.

## Conclusion

The COVID-19 pandemic has exposed weaknesses in the Swiss health care system, that existed before and were exacerbated during the crisis. Many recommendations identified in this study are beneficial to patients and caregivers in general and should be implemented regardless of a possible future pandemic, for example, the use of technology for remote consultations, vaccinations at home, and more time for the professional care of elderly or people with cognitive impairments. These implementations will increase the systemic resilience of the health care system and decrease the burden of individuals and the communities affected by dementias.
